# Secondary Cutaneous Endometriosis of the Umbilicus in Tanzania: A Case Report

**DOI:** 10.24248/eahrj.v7i2.719

**Published:** 2023-11-30

**Authors:** Isaac H. Makanda, Beata N. Mushema, Salvatory P. Chuwa, Monica Chiduo

**Affiliations:** a Department of Obstetrics and Gynaecology, Hubert Kairuki Memorial University, Dar es Salaam, Tanzania

## Abstract

**Introduction:**

Endometriosis is characterised by endometrial tissue outside the endometrial cavity. The implantation sites may be pelvic or extrapelvic in nature. Umbilical endometriosis is a rare type of cutaneous endometriosis, accounting for 0.5–1% of extrapelvic endometriosis cases. Current literature on umbilical endometriosis is absent in the Tanzanian population.

**Case Report:**

A 30-year-old woman with prior caesarean deliveries presented with a 3-year history of umbilical swelling, cyclical pain, and menses-related bleeding. Examination revealed a firm brown umbilical lesion measuring 5×4 centimetres (cm). Wide excisional biopsy was performed. Histopathological examination confirmed endometriosis and ruled out malignancy. The postoperative follow-up revealed no signs of recurrence.

**Conclusions:**

This case report highlights the need for high clinical vigilance and comprehensive differential diagnosis, especially for recurring and cyclical abdominal symptoms. Despite resource limitations, accurate diagnosis and appropriate treatment can result in the successful management of this rare condition. The report emphasises the urgency for clinicians to boost awareness, promote research, and advocate for better resources to ensure optimal patient outcomes.

## INTRODUCTION

Endometriosis is a clinical entity characterised by the implantation of endometrial glands and stroma outside the endometrial cavity. The exact prevalence of this condition varies according to the study population, design, setting, and diagnostic approaches. However, according to prevalence estimates, endometriosis affects as many as 10% of premenopausal women worldwide.^1,2^ The prevalence of endometriosis among indigenous African women of reproductive age is between 2% and 10%. While there is no specific prevalence data on Tanzania, this suggests that the prevalence of endometriosis in Africa is generally low.^3^

Although pelvic sites of endometriosis such as; ovaries, uterosacral ligaments, Pouch of Douglas, sigmoid colon, rectum, and the urinary bladder are relatively more common, there have been cases in literature of extra-pelvic endometriosis located on surgical scars, pleura, diaphragm, pericardium, nasal cavity, liver, kidneys, nerves, and muscles.^4–18^

Umbilical endometriosis, a form of cutaneous endometriosis accounts for 0.5 to 1% of extra-pelvic cases of endometriosis and 30 to 40% of abdominal wall endometriosis.^19,20^ It is classified as either primary or secondary; the latter is more common and associated with prior surgical procedures and the former is seen among patients without prior history of surgery. Patients typically present with cyclic pain, bleeding, and swelling related to menstrual cycle.

The diagnosis of umbilical endometriosis is largely clinical and imaging studies have a limited role. It is worth noting that umbilical endometriosis may be confused with other conditions, such as; Sister Mary Joseph nodule, hernia, melanoma, and keloids, which can delay diagnosis and appropriate management.^21^ The definitive treatment is wide local excision of the lesion. Hormonal treatment may be used to relieve symptoms and reduce the size of the mass preoperatively but has no curative role. Histopathological examination of tissue confirms endometriosis and excludes malignancy.

In this study, we describe a case of secondary umbilical endometriosis in an East African setting. This case is notable not only because of the relative rarity of this condition, but also because it underscores the importance of heightened clinical suspicion, especially in the face of prior abdominal surgeries and unresponsive treatments. This highlights the value of correct diagnosis and demonstrates how effective management can result in successful outcomes, even in low-resource settings. By bringing attention to this uncommon condition, we hope to stimulate further research on endometriosis within this region, increase awareness among healthcare professionals, and underline the need for improved diagnostic and treatment resources.

## CASE REPORT

**FIGURE 1:**
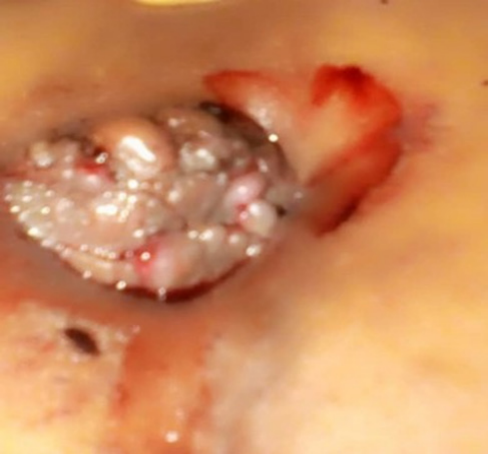
Umbilical Swelling During Menses

A 30-year-old multiparous woman presented to our tertiary health facility with a history of cyclical painful umbilical swelling for the past 3 years. The pain was severe to the extent that it interfered with her daily activities and was noted only during her menses. Initially, the swelling did not bleed but started to bleed after 1 year (Figure 1). She also reported experiencing cramping abdominal pain about 2 days before menses, as well as a tingling and itching sensation of the swelling towards the end of her menses. Prior to arriving at our facility, she had attended 4 other health facilities, where she was misdiagnosed as having keloids and treated with corticosteroid injections with no relief of her symptoms.

She had a history of caesarean deliveries with a subumbilical midline incision in 2010 and 2014 due to a contracted pelvis. Her past menstrual history was unremarkable. Menarche was attained at 14 years of age, and she had a regular 28-day cycle not accompanied by dysmenorrhoea. Her medical, gynaecological, and social history was unremarkable.

**FIGURE 2:**
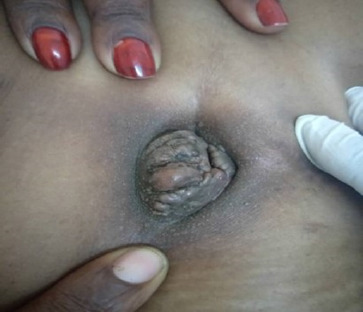
Umbilical Swelling on Examination

Initial physical examination was performed when the patient was not in her menses and revealed a brown, firm, multilobulated nodule measuring 5 × 4 cm. The nodule was tender on deep palpation and did not bleed upon contact. The surrounding area was hyperaemic, approximately 1 cm from the nodule/mass (Figure 2).

**FIGURE 3:**
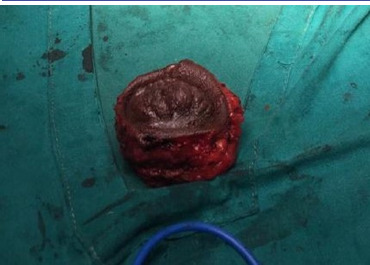
Excised Umbilical Lesion

The clinical diagnosis of umbilical endometriosis was made based on the patient's history and examination findings. The patient underwent wide excisional biopsy of the mass under general anaesthesia. A diamond-shaped incision was made around the mass, with margins extending to the surrounding hyperaemic area. The incision extended down to the peritoneum and the entire mass was excised with clean margins (Figure 3). The incision was closed in layers and the patient received postoperative management according to the hospital protocol. The sutures were removed on the 7^th^ postoperative day.

**FIGURE 4:**
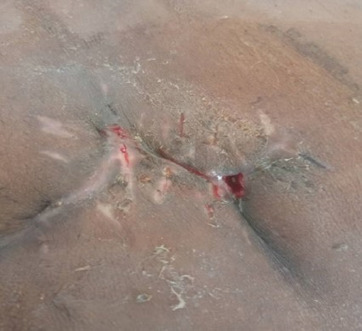
Surgical Site at Follow Up

The biopsied tissues were sent for histopathological analysis. Gross examination revealed a firm greyish-white mass with areas of congestion. Histology revealed connective tissue with endometrial tissue and a few glands without a complex branching pattern. Inflammatory cells surrounded the endometrial tissue. There was no evidence of metastasis or malignancy (Figure 4).

Follow-up of the patient at approximately 3 months postoperatively showed complete cessation of symptoms and no signs of recurrence.

### Ethical Approval

Ethical clearance to publish this case report was granted by Kairuki Hospital (KH/IM/HKMU/7/2021).

### Consent

Written informed consent was obtained from the patient for the procedure, publication of this case report, and the accompanying images.

## DISCUSSION

Endometriosis is an increasingly common gynaecological disorder with potential to affect social and psychological quality of life of affected individuals. It may be considered as a multi-systemic condition, owing to the numerous sites of implantation of endometrial tissue, whether pelvic or extra-pelvic.^22^ The prevalence of endometriosis is highly varied and the current literature is lacking among the Tanzanian population.^19,23–25^

The study candidate was of childbearing age and presented with umbilical swelling associated with cyclical pain and bleeding, which is consistent with the literature.^22,26^ Her condition occurred for 3 years before the correct diagnosis was made, which is considerably later than the average time-to-diagnosis reported in studies conducted elsewhere.^19,21,26^ This is most likely due to the fact that her condition was first misdiagnosed and managed as keloid in health facilities elsewhere.

Local examination of the swelling in our case revealed a diameter of 5 cm × 4 cm. This is larger compared with what is reported in other literature.^19,21,26,27^ The diagnosis of umbilical endometriosis was clinical in our case and supported by history of lack of response to corticosteroid injections for keloid, as was in similar reports.^19,26^

Our patient underwent wide local excision of the mass, and histopathological examination confirmed endometriosis and ruled out signs of malignancy. Three-month follow up showed complete resolution of symptoms and no evidence of recurrence. This treatment approach and outcome were similar to those reported in other related studies.^19,21,24^ Some studies recommended additional laparoscopic pelvic observation, as up to 15% of cases of umbilical endometriosis occur with pelvic endometriosis. However, in our case this was not performed and might not be feasible in low-resource settings where laparoscopy surgeries are not performed in majority of health facilities.^26–28^

Although extrapelvic endometriosis is thought to be rare, there have been increasing reports of these cases in various settings.^22^ This case report underscores the importance of heightened clinical vigilance and broad differential diagnosis, particularly in cases presenting with recurring, cyclical abdominal symptoms, such as pain or swelling.

## CONCLUSION

The successful management of this rare case illustrates that, with accurate diagnosis and appropriate treatment, even complex conditions can be effectively addressed in resource-limited settings. It is crucial that clinicians improve awareness, foster research in underrepresented regions, and push for enhanced diagnostic and treatment resources to ensure optimal patient outcomes.
